# Cytotoxicity of orthodontic temporary anchorage devices on human periodontal ligament fibroblasts in vitro

**DOI:** 10.1002/cre2.230

**Published:** 2019-08-08

**Authors:** Zhibin Chen, Manika Patwari, Dawei Liu

**Affiliations:** ^1^ Department of Periodontology Peking University School of Stomatology Beijing P.R. China; ^2^ Private Practice, Ridgeview Dental Group Menomonee Fall Waukesha County Wisconsin; ^3^ Department of Developmental Sciences/Orthodontics, School of Dentistry Marquette University Milwaukee Wisconsin

**Keywords:** cytotoxicity, orthodontics, temporary anchorage device (TAD)

## Abstract

**Objectives:**

The objective of this study is to test cytotoxicity of four brands of commercially available orthodontic temporary anchorage devices (TADs).

**Setting and sample population:**

Twenty‐four (six for each brand, i.e., Aarhus [AO]; Dual top [RMO]; Vector TAS [ORMCO]; and Unitek TAD [3M UNITEK]) TADs were tested.

**Materials and methods:**

Twenty‐four (six for each brand, i.e., Aarhus [AO]; Dual top [RMO]; Vector TAS [ORMCO]; and Unitek TAD [3M UNITEK]) TADs were individually incubated in complete cell culture medium and shaken at a rate of 1.5 rpm at 37°C for 30 days to extract possible toxic substances in conditioned media (CM). To test cytotoxicity, human periodontal ligament fibroblasts were cultured and exposed to the CM for 24 hr, followed by the examinations of morphological changes, cell viability (MTT assay), and cell damage (lactate dehydrogenase [LDH] assay).

**Results:**

No morphological changes were observed in any of the four brands of TADs compared with the negative control. LDH assay showed that none of the four brands of TADs caused significant cell damage after CM treatment compared with the negative control (*P* > .05). No significant differences were found between any of the four brands of TADs (*P* > .05). MTT assay showed similar results as did the LDH assay, except for a statistically significant difference found in the TADs from 3M UNITEK compared with the negative control (*P* = .047).

**Conclusions:**

According to the International Standard Organization standards, except for the TAD from 3M, none of the other three brands of commercially available TADs (from AO, RMO, and ORMCO) exhibited significant cytotoxicity, suggesting their safe clinical applications.

## 1 INTRODUCTION

The idea of using bone screws in orthodontics dates back to 1983, when Creekmore and Eklund firstly reported the use of a Vitallium bone screw to treat a patient with deep bite. In 1997, Kanomi described a mini implant specifically made for orthodontic use(Kanomi, 1997), and in 1998, Costa et al. presented a screw with a bracket‐like head(Costa, Raffainl, & Melsen, 1998). Ever since, rapid developments have ensued in using temporary anchorage devices (TADs) to gain skeletal anchorage. TADs are now used for a plethora of orthodontic movements including correction of deep bite, space closure, correction of asymmetric cant, molar extrusion and intrusion, distalization, mesialization, and en‐masse retraction, to name a few (Papadopoulos & Tarawneh, 2007; Reynders, Ronchi, & Bipat, 2009; Shirck, Firestone, Beck, Vig, & Huja, 2011; Wahl, 2008; Yamaguchi, Inami, Ito, Kasai, & Tanimoto, 2012; Yanosky & Holmes, 2008). With their smaller size, wider applications in tooth movements, simpler surgical placement, and immediate loading, TADs have become a mainstay in contemporary orthodontics.

A successful material to be used as for TADs should be biocompatible and have good mechanical properties and corrosion resistance. Most commercially available orthodontic mini implants or TADs are made of titanium alloys, primarily Ti‐6Al‐4V(Reynders et al., 2009). Titanium has the property of oxidizing in the presence of air and aqueous electrolytes to form a passive titanium dioxide film that contributes to its biocompatibility and corrosion resistance(Velasco‐Ortega, Jos, Cameán, Pato‐Mourelo, & Segura‐Egea, 2010) but needs to be alloyed to improve its strength and fatigue resistance(Eliades, Zinelis, Papadopoulos, & Eliades, 2009). The titanium alloy is composed of a fusion of two phases, alpha (6% aluminum) and beta (4% vanadium). Both phases in equilibrium contribute towards advantages of mechanical resilience (alpha phase), good formability, and fatigue resistance (beta phase)(Cotrim‐Ferreira, Quaglio, Peralta, Carvalho, & Siqueira, 2010). However, this leads to a decrease in the corrosion resistance of the Ti alloy in body fluids. Ti‐6Al‐4V alloys used in orthopedics for joint replacements have shown to be susceptible to bio‐corrosion in the physiological environment of the human body(Cadosch et al., 2010; Cadosch, Chan, Gautschi, & Filgueira, 2009; David & Lobner, 2004; Knutson & Berzins, 2013). By the same rationale, TADs composed of Ti‐6Al‐4V alloys would be susceptible to bio‐corrosion despite their shorter duration of use. De Morais et al. proposed that TADs are a potential source of metallic ions to the human body because of the corrosion of titanium (Ti) alloy in body fluids. They evaluated the systemic levels of metallic ions specifically the concentrations of titanium (Ti), vanadium (V), and aluminum (Al) in rabbits. Low amounts of Ti, Al, and V were detectable in the 1‐, 4‐, and 12‐week groups of the rabbits, confirming that release of these metals from the mini implants occurs, with diffusion and accumulation in remote organs such as kidneys, liver, and lungs. However, despite the tendency of ion release when using the Ti alloy as TADs, the amounts of metals detected were significantly below the average intake of these elements through food and drink and did not reach toxic concentrations(de Morais et al., 2009). Whether the released metal ions cause damage to the local cells or not remains unknown, which asks for more studies, for example, basically cytotoxicity to be done.

Despite its routine clinical use, current literature detailing cytotoxicity of TADs is rare. A recent study tested cytotoxicity of orthodontic mini implants and found that orthodontic mini implants were not cytotoxic to human gingival fibroblasts but cytotoxic to mouse osteoblasts(Malkoç, Öztürk, Çörekçi, Bozkurt, & Hakki, 2012). With the increasingly rampant applications of TADs, it becomes critical to address this important issue of potential cytotoxicity.

Based on the previous studies mentioned above, the aim of this study was to test the cytotoxic effects of TADs on human periodontal ligament fibroblast (hPDLF) cells in vitro. To test the cytotoxicity, we subjected the hPDLF cells to the conditioned media (CM) collected after 30 days of incubating TADs in complete cell culture media, followed by morphological observations as well as cell viability test using MTT assay and cell damage test using lactate dehydrogenase (LDH) assay.

## 2 MATERIALS AND METHODS

In accordance with the International Standard Organization (ISO) standards(International Standard Organization, 2009), six of each of the four brands of commercially available TADs (brand name, company; Unitek, 3M UNITEK; Aarhus, AO; Vector TAS, ORMCO; and Dual Top, RMO) were used in this study. All the TADs were tested directly from their sterile surgical packages as received, except for the product from AO that was autoclaved prior to use due to its unspecified sterile condition. According to the ISO standards (International Standard Organization, 2009), cytotoxicity can be tested by direct contact or extraction means. Due to the complicated surface topography of TADs and our purpose of stringently challenging the release of the possible toxic substances, we chose to use the extraction method, that is, to incubate TADs in cell culture medium for certain time to extract possible toxic substances and collect the CM for cytotoxicity test. Another advantage of using extraction method is to be able to extract the possible toxic substances for a long period of time as reported previously(Malkoç et al., 2012).

**Figure 1:**
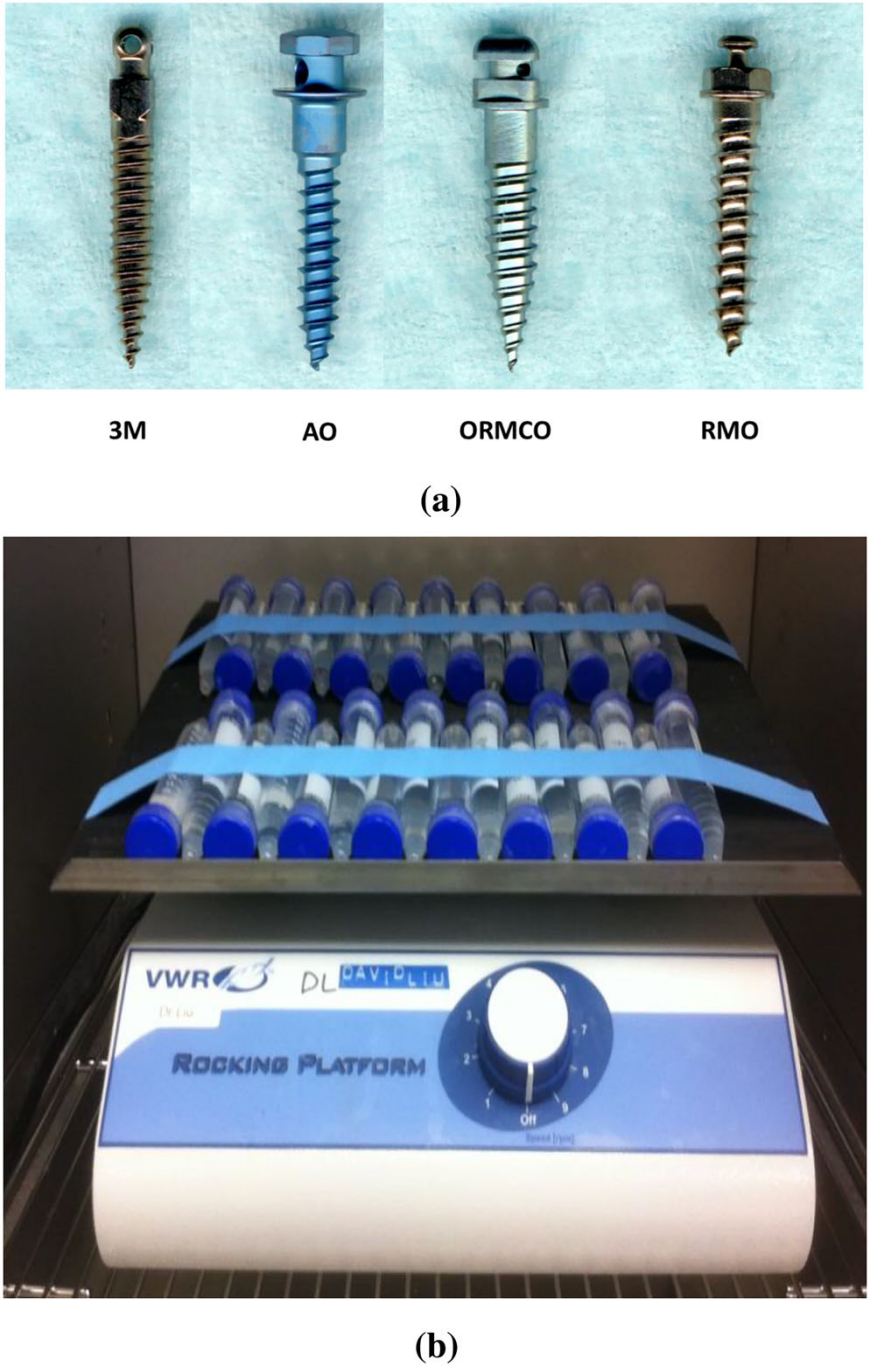
Experimental setup. (a) Sample pictures of the TADs tested in this study and (b) the experimental setup for incubating the TADs in cell culture media to extract potential toxic substances on shaker (1.5 rpm) at 37°C for 30 days. TADs, temporary anchorage devices

Twenty‐four TADs (six for each brand) were individually submerged in 8‐ml alpha‐minimal essential medium supplemented with 10% fetal bovine serum and 1% penicillin and streptomycin (10,000 units of penicillin and 10 mg of streptomycin in 0.9% NaCl) and sealed in 15‐ml volume test tubes. To mimic the clinical intraoral environment where the TADs are placed and exposed to the flow of saliva(Dawes, 2008), the test tubes were constantly shaken at a rate of 1.5 round per minute (rpm)(Zhou, Liu, You, & Wang, 2010) at 37°C in the cell culture incubator (Figure 1) for 30 days (the minimal duration of clinical use of TAD in orthodontics) to stringently trigger the release of the possible toxic substances from the TADs. By the end of 30 days, the CM was collected for cytotoxicity tests. In addition to the experimental groups, a control group (*n* = 6) was set and underwent the same experimental procedures but without TADs involvement.

PDLF are the most abundant cells in the PDL, which have been extensively used in dental studies. According to the ISO 10993‐5 guidelines(International Standard Organization, 2009), cell lines are recommended to use in the cytotoxicity test. Therefore, in this study, a commonly used human PDLF cell line #2630 (ScienCell Research Laboratories, Inc. Carlsbad, CA) was chosen. The cells were cultured in the same type of cell culture medium as used for incubating TADs in a humidified atmosphere of 5% CO_2_, 95% air at 37°C. To set for cytotoxicity, the cells were seeded at a density of 5 × 10^4^ cells/ml/well in 24‐well plates for 24 hr and then treated with the CM for 24 hr. As a positive control, 0.1% Triton X‐100 (Sigma, St. Louis, MO) was used to generate cell damage and death, whereas the cells under the treatment of CM without TADs were used as a negative control. After 24 hr of treatment, morphological changes as well as cell damage and viability were examined.

The cell shape and size were observed under the microscope (Nikon Eclipse Ti‐S, Nikon Instruments Inc, America), and digital images were taken for all the groups under the magnification of 20×.

LDH is an enzyme located in the cytosol and is released into culture medium upon cell damage or lysis. LDH activity in the culture medium can thus be used as an indicator of cell membrane integrity and hence of cytotoxicity(David & Lobner, 2004; Haslam, Wyatt, & Kitos, 2000; Wolterbeek & van der Meer, 2005). In this study, the quantity of LDH release after treating the cells with the CM for 24 hr was determined following the assay protocol of Cayman LDH Cytotoxicity Assay Kit (Cayman chemical company, Ann Arbor, MI). The absorbance was read at 490‐nm wavelength with a plate reader (Bio‐Tek power wave XS2, Winooski, VT). Blank LDH levels were subtracted from insult LDH values.

The MTT assay is based on the measurement of cell viability via metabolic activity. As a reagent, yellow water soluble MTT (3‐(4,5‐dimethylthiazol‐2‐yl)‐2,5‐diphenylytetrazoliumbromid) is metabolically reduced in viable cells to a blue–violet insoluble formazan. The number of viable cells correlates with the color intensity determined by photometric measurements(Scudiero et al., 1988; Sjögren, Sletten, & Dahl, 2000). The reduction of MTT is thought to occur mainly in the mitochondria through the action of succinate dehydrogenase, therefore providing a measurement of mitochondrial function. The hPDLF cell damage was thus quantified by measurement of the reduction of MTT to produce dark blue formazan crystals in accordance with the test protocol of the MTT Cell Proliferation Assay Kit (Cayman chemical company, Ann Arbor, MI). To perform the measurement, 75‐μl solution MTT was added at the end of the treatment (24 hr), and after 3 hr of incubation, the medium was removed and the resulting MTT formazan crystals were dissolved by the addition of MTT solvent. The assay of the formation of formazan was performed by measuring the amount of reaction product by absorbance change using the microplate reader at a wavelength of 570 nm.

All the MTT and LDH assay data were expressed as means ± standard deviation (*n* = 6) in the graphs. Statistically, one‐way analysis of variance was used to determine the difference between all the experimental groups as a whole and the controls, whereas Bonferroni post hoc adjustment was applied to find out the difference between any two of the four TAD groups. A *P* value less than.05 was considered significant (SPSS, version 11.10, Chicago, IL).

## 3 RESULTS

**Figure 2:**
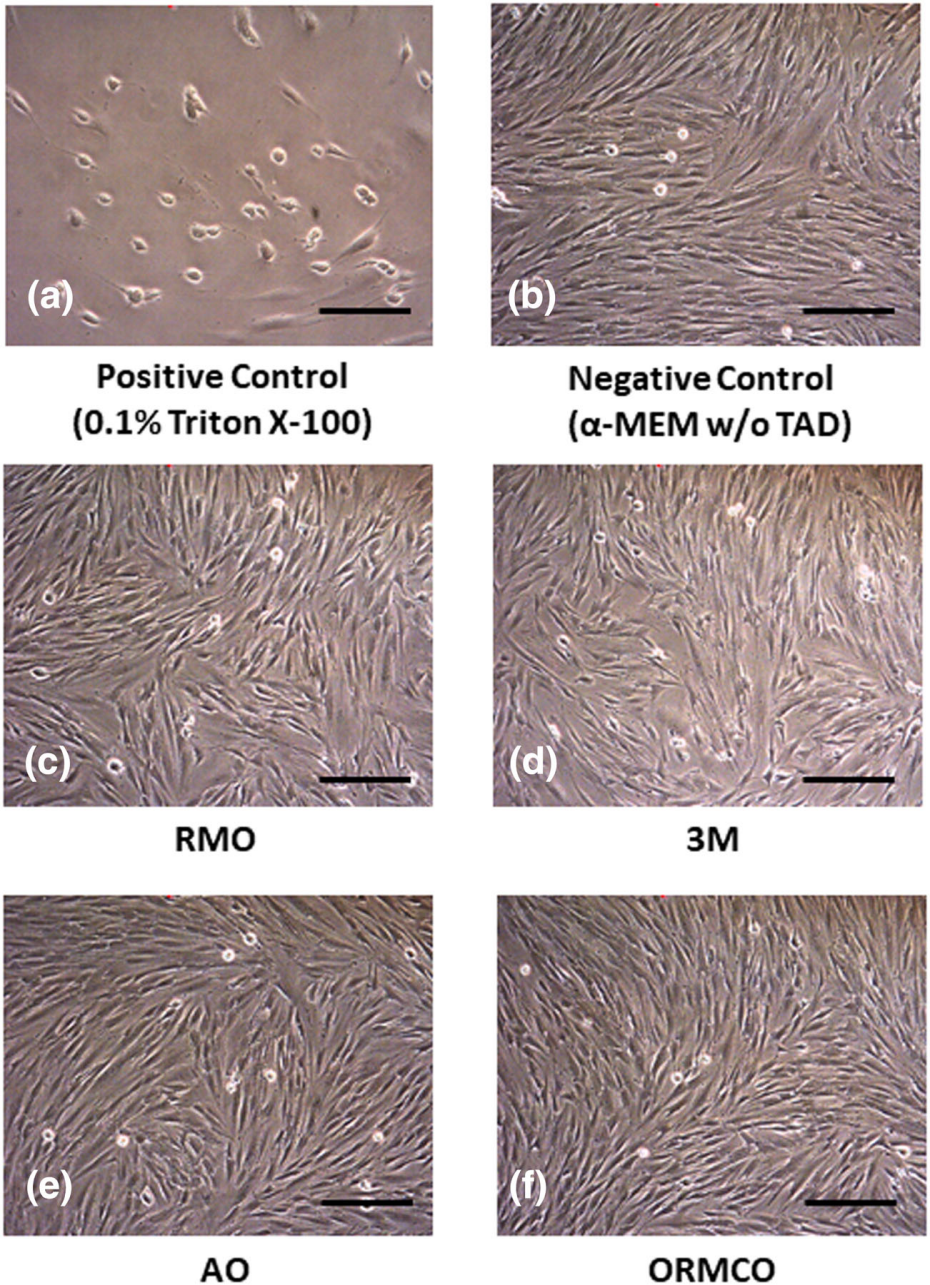
Morphological changes of hPDLF cells after exposure to CM for 24 hr. In comparison with the positive control, none of the TADs groups showed significant changes of cell shape, size, and orientation, which is similar to the negative control (Bar = 40 μm). α‐MEM, alpha‐minimal essential medium; CM, conditioned media; hPDLF, human periodontal ligament fibroblasts; TADs, temporary anchorage devices

Morphologically, the cells in all the TADs groups (Figure 2c–f) under CM treatments were spindle shaped, not observably different from each other, and similar to the cells in the negative control group (Figure 2b). However, the cells under the treatment of 0.1% Triton X‐100 were round instead of spindle shaped, lost their cellular orientation, and mostly detached from the field (Figure 2a).

**Figure 3:**
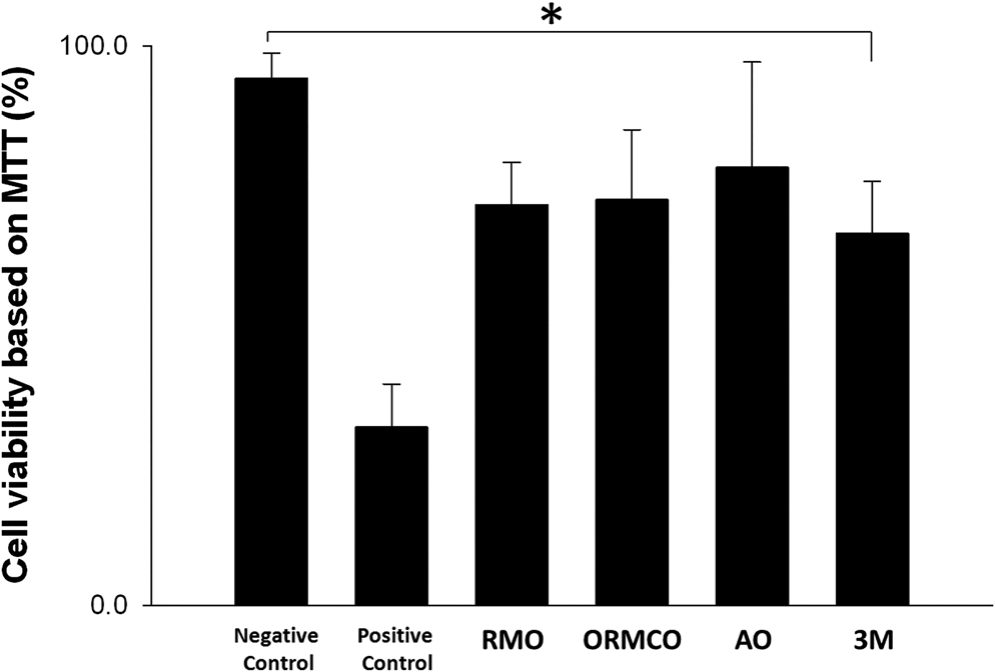
MTT assay‐based cell viability of hPDLF cells after exposure to CM for 24 hr. In comparison with the negative control, three TADs groups (RMO, ORMCO, and AO) showed no significant reduction of cell viability (*P* > .05, *n* = 6), opposite to the positive control showing a 66% cell viability reduction (*P* < .05, *n* = 6). However, the TADs from 3M UNITEK exhibited a statistically significant 30% reduction of cell viability when compared with the negative control (*P = .047, *n* = 6). CM, conditioned media; hPDLF, human periodontal ligament fibroblasts; TADs, temporary anchorage devices

The MTT cytotoxicity test quantitatively measures the cell viability. Our positive control group (exposed to 0.1% Triton X‐100) had the lowest MTT value (0.16 ± 0.039), whereas the negative control (treated with CM without TADs) had the highest MTT level (0.47 ± 0.023). There was a highly significant difference between the positive control group and all the other groups (*P* = .000). There was no significant difference among the different brands of TADs (*P* > .05), except that the 3M Unitek product showed a statistically significant cytotoxicity (lost cell viability by about 30%) when compared with the negative control group (*P* = .047; Figure 3).

**Figure 4:**
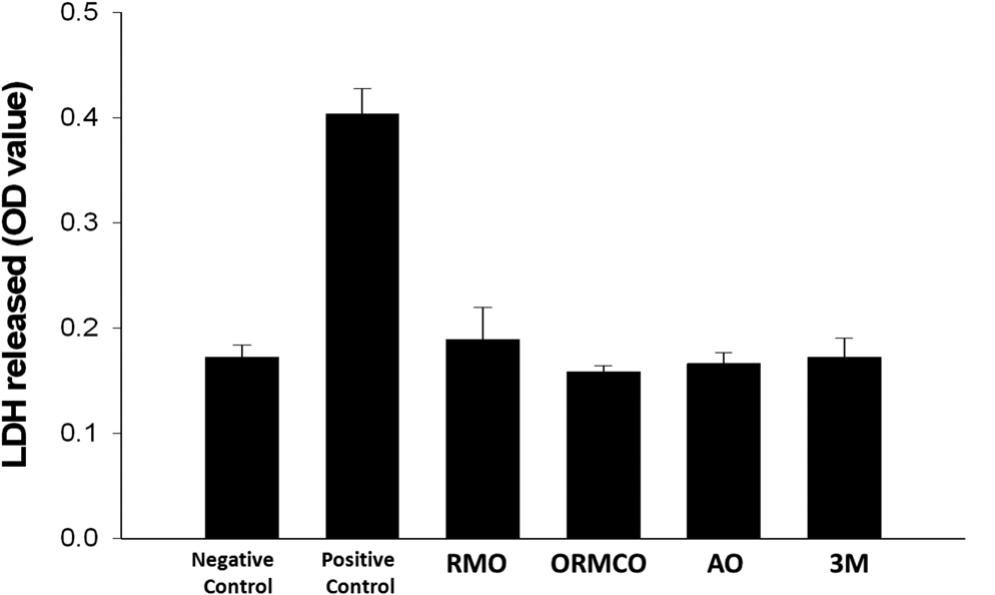
LDH release from hPDLF cells after exposure to CM for 24 hr. In comparison with the negative control, all four TADs groups (RMO, ORMCO, AO, and 3M) showed no statistically significant increase of LDH (*P* > .05, *n* = 6), opposite to the positive control showing a 1.35‐fold increase of LDH (*P* < .05, *n* = 6). CM, conditioned media; hPDLF, human periodontal ligament fibroblasts; LDH, lactate dehydrogenase; TADs, temporary anchorage devices

When the cellular plasma membrane is damaged or upon cell lysis, LDH is released from the cells, which is used as an indicator of cell membrane integrity and a measurement of cytotoxicity(David & Lobner, 2004). In our study, the positive control group cells treated with 0.1% Triton X‐100 showed the highest release of LDH (0.40 ± 0.024) and thus the highest cytotoxicity, whereas the negative control resulted in the lowest LDH value (0.17 ± 0.012). All the four TADs groups together with the negative control showed significantly low LDH levels than that in the positive control (*P* = .000). There was no significant difference in LDH levels among the four different groups of TADs (*P* > .05; Figure 4).

## 4 DISCUSSION

Biosafety and biocompatibility are of main concern to the clinical application of dental materials including TADs. in vitro cytotoxicity tests are advised by ISO to evaluate acute cytotoxicity of a material(International Standard Organization, 2009) and also aid in better understanding the pathogenicity of subacute effects. In contrast to animal experiments, cell cultures commonly used for dental material biosafety tests are generally simple, inexpensive, and can be performed under well‐controlled conditions(Mockers, Deroze, & Camps, 2002; Samara et al., 2011). Ideally, cytotoxicity tests should be done on the same type of tissue that the tested compounds will be exposed to, and efforts should be made to simulate in vivo conditions as much as possible. Although many types of cells (primary cells vs. cell lines) can be used in the cytotoxicity test, it is recommended by ISO that cell lines should be used instead of primary cells because the established cell lines are morphologically and physiologically more homogenous and thus can produce reliable and reproducible results despite their difference from the primary cells(Hernández‐Sierra et al., 2011). Therefore, we chose the popularly used hPDLF cell line #2630 as the cell source in this study.

In this study, we used the commercially available TADs rather than Ti‐6Al‐4V discs(Watanabe et al., 2004), because it is more clinically relevant to use the products that are used intraorally. In addition, we submerged the TADs in the complete cell culture medium for 720 hr (30 days) on a flip‐flop shaker (at a rate of 1.5 rpm) to mimic in vivo conditions favoring bio‐corrosion and stringently stimulate the release of possible toxic substances (mainly metal ions) over a long period of time. In accordance with ISO guidelines, cytotoxicity test can be done in two ways—contact (direct) and extract (indirect). Due to the complex surface of the TADs and the limitations of direct method (long time culture cannot be performed), we chose to use the extract means. We incubated the TADs in cell culture medium for 30 days, much longer than the 24–72 hr as recommended in ISO standards in an attempt to make a stringent extraction of the possible toxic substances from the TADs. If long time extracts are not cytotoxic, it thereby follows that the clinical application in shorter time period should be safe.

In this study, no significant cytotoxic effects were found among the tested TADs for the morphological changes of the cells and the LDH assay analysis. This is not surprising as all these TADs have similar composition (Ti‐6Al‐4V). MTT assay analysis showed that among the four TAD groups, the 3M Unitek TADs had a 30% reduction of cell viability when compared with the negative control (*P* = .047). The explanation to this may lie in further evaluating bio‐corrosion. A recent study examined the corrosion of TADs from 3M Unitek, ORMCO, and AO in artificial saliva and found subtle but not significant differences in the passivity of all the TADs tested. The 3M Unitek TADs had a comparatively less stable passive layer at open circuit potential above 0.3V(Knutson & Berzins, 2013), and a less stable passive oxide layer typically is associated with greater corrosion rate(Bohni, 2005). The authors also observed that the silver/grey colored Unitek TADs may suggest a thinner oxide layer in contrast to the AO and ORMCO TADs that were blue and pink colored. Different surface treatment may or may not have been performed on the TADs accounting for the variability in oxide layer. However, it is of value to consider that some manufacturers provide color‐coded options to differentiate sizes and locations for use and so not all TADs from a particular manufacturer may perform the same(Knutson & Berzins, 2013). In contrast to our study, both 3M Unitek and RMO products had similar color but 3M Unitek TADs still had a slightly less cell viability based on MTT assay analysis. The AO TADs with the blue color had a significantly more noble open circuit potential (*P* < .05) compared with the others(Knutson & Berzins, 2013). Although our TADs were submerged in culture medium instead of artificial saliva, the same medium was used for all test products, and hence, this additive variable was eliminated in our study.

Despite similar composition, TADs have shown to cause variable cellular reactions on different cell types in previous studies. In another in vitro study, Malkoc et al. observed that the same Ti‐6Al‐4V alloy in MTN (Turkey) and Vector TAS (ORMCO) significantly decreased the MC3T3‐E1 (mouse osteoblasts) cell viability at 190 hr in contrast to Abso Anchor (Dentos, Incorporated, Dong‐In‐Dong Jung‐Gu Daugu, South Korea) and IMTEC Ortho (3M Unitek, Europe). None of the TADs had significant adverse effects on human gingival fibroblasts (Malkoç et al., 2012). Velasco‐Ortega et al. found that Ti‐6Al‐4V discs pretreated by a nitric acid passivation process were nontoxic to human or mouse fibroblasts. They noted that passivation will lead to a more dense stable oxide layer over the alloy surface and hence increase corrosion resistance(Velasco‐Ortega et al., 2010). Interestingly, another study done on MC3T3‐E1 cells in contact with titanium alloy discs (Ti‐6Al‐4V) reported a transient reduction in their cell viability at Day 4. This decrease was restored by Day 8 and completely eliminated after 15 days (360 hr) in culture. The authors attributed this transient alteration of cell viability to the chemical composition (Citeau et al., 2005). Okazaki et al. observed decreased growth ratio of MC3T3‐E1 cells around Ti‐6Al‐4V alloys than in the presence of pure titanium and suggested that it was because of toxic effects of released vanadium ions(Okazaki, Rao, Ito, & Tateishi, 1998). The reasons of different responsiveness of different types of cells (e.g., osteoblasts vs. fibroblasts) and of same type of cells but from different species (e.g., human vs. mouse) are unknown.

There were limitations in this study. It is essential to appreciate the factors associated with cytotoxicity results namely dose of the toxin, exposure time, cell type, and mechanism of action (necrosis vs. apoptosis). Even though the ISO standards were followed in this study as in others, special attention needs to be paid when attempting to interpret the results and relate them to clinical situations. Considering the variables of difference in methodology (discs vs. TADs, end point testing vs. real cell analysis, incubation, and testing duration) and different cell origins, it is expected to see variable outcomes. Further standardization of cytotoxicity tests needs to be considered in order to draw more reliable and reproducible results. The release of metal ions from TADs might directly affect their biocompatibility. There are no exhaustive data correlating metal ion release from TADs, their biocompatibility, and association with failure of orthodontic mini implants or TADs.

Considering the clinical importance of TADs in orthodontics, further investigations should be performed to better understand the biological effect of the TADs on oral tissues.

## 5 CONCLUSIONS

Within the limitations of this in vitro study, according to the ISO standards, except for the TAD from 3M, none of the other three brands of commercially available TADs (from AO, RMO, and ORMCO) exhibited significant cytotoxicity, suggesting their safe clinical applications.
